# Supplementary figures and images for: APEX-based proximity labeling in Plasmodium identifies a membrane protein with dual functions during mosquito infection
Source: PLoS Pathog. 2024 Dec 18;20(12):e1012788. doi: 10.1371/journal.ppat.1012788 (PMC11695019; doi:10.1371/journal.ppat.1012788)

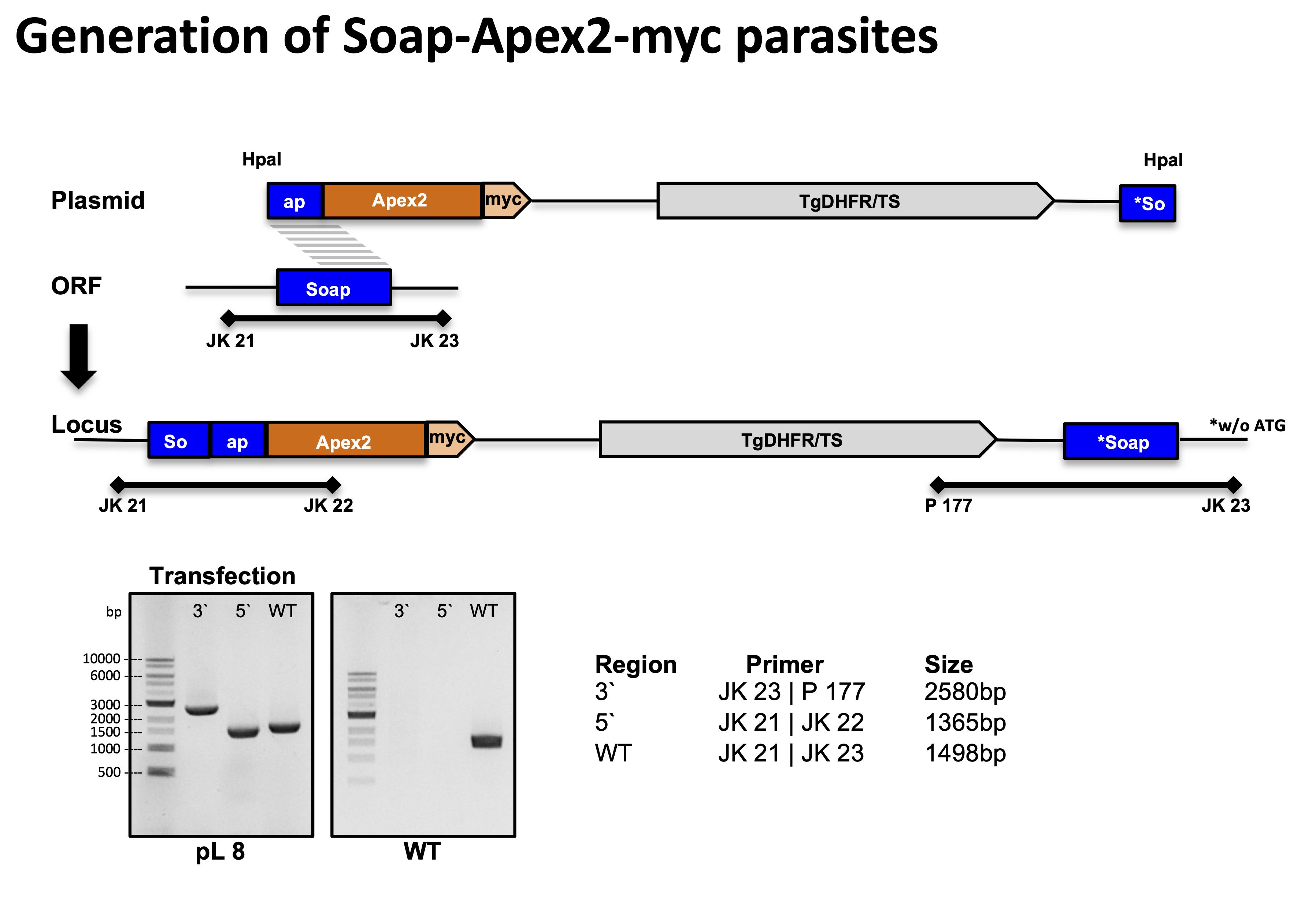

Supplement: S1 Fig — The cartoon shows the cloning strategy and primers used for genotyping with amplicon sizes of the resulting transgenic line indicated. For primer sequences please see S2 Table. Plasmid contains as resistance marker the dehydrofolatereductase/thymidine-synthase from Toxoplasma gondii (Tgdhfr/ts) (grey). Note that the second copy of soap lacks the ATG and should not be expressed. (TIFF) [file ppat.1012788.s001.tiff]

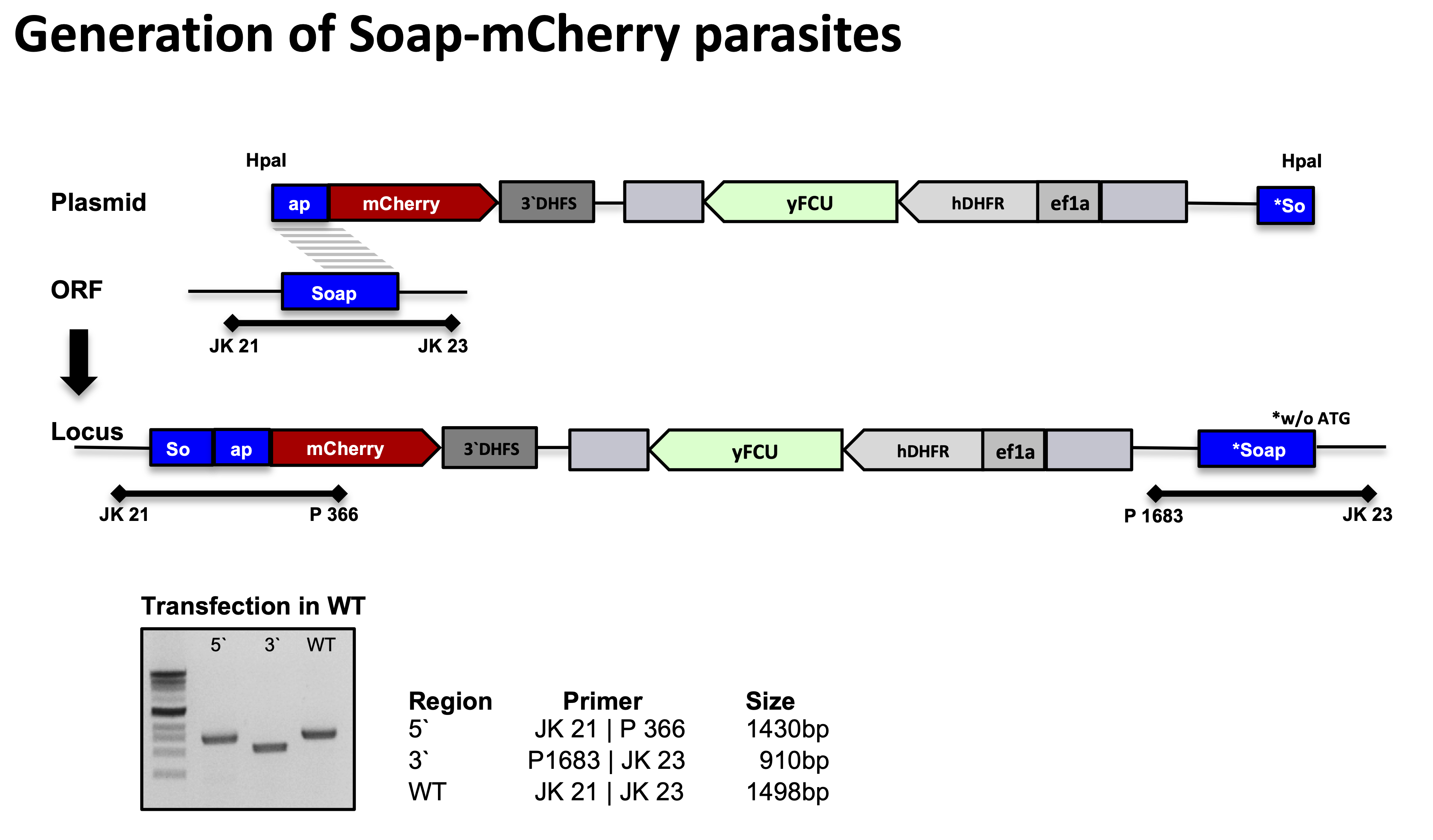

Supplement: S2 Fig — The cartoon shows the cloning strategy and primers used for genotyping with amplicon sizes of the resulting transgenic line indicated. For primer sequences please see S2 Table. Plasmid contains as resistance marker the dehydrofolatereductase/thymidine-synthase from Toxoplasma gondii (Tgdhfr/ts) (grey). Note that the second copy of soap lacks the ATG and should not be expressed. (TIFF) [file ppat.1012788.s002.tiff]

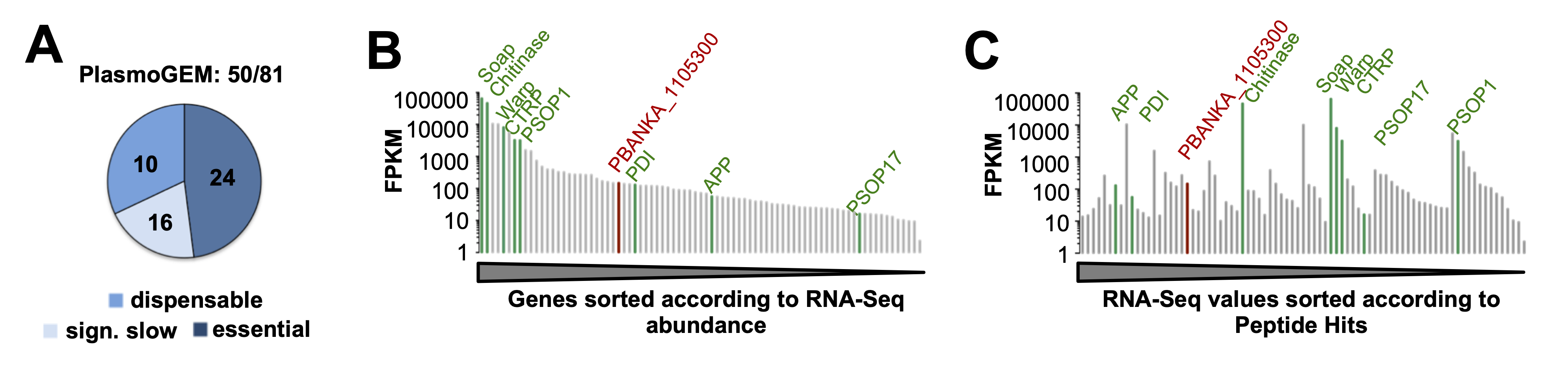

Supplement: S3 Fig — (A) 50 of the 81 micronemal candidate proteins were already investigated by the PlasmoGEM screen of which 24 were classified as essential, 16 conferred slow growth upon deletion and 10 were dispensable. (B-C) RNA-seq abundance (y-axis) of the identified micronemal candidate proteins [44] with known proteins (green) and the top candidate, PBANKA_1105300 (red) marked sorted (x-axis) according to RNA-seq data (B) and by peptide abundance from our BioID screen (C). (TIFF) [file ppat.1012788.s003.tiff]

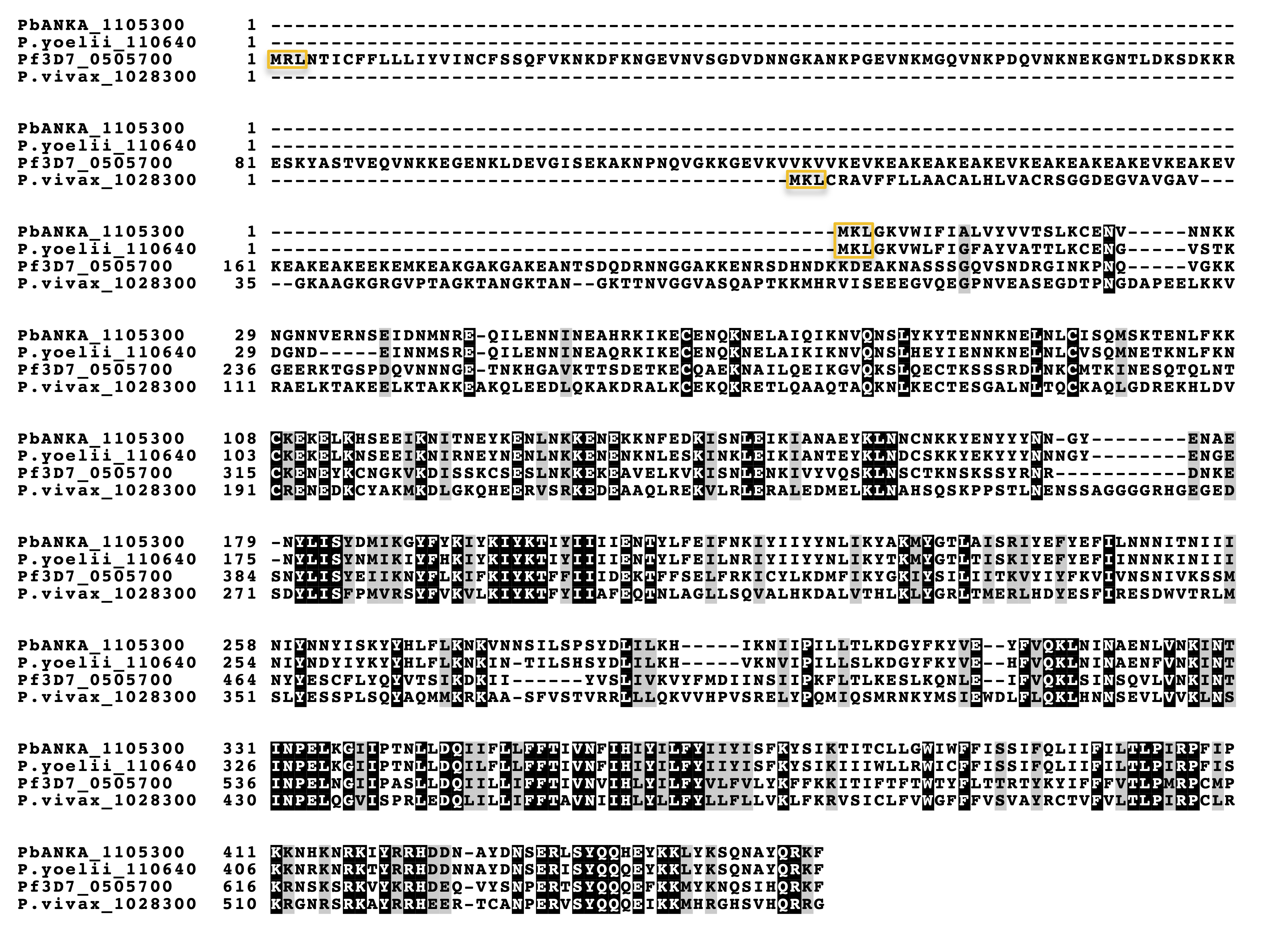

Supplement: S4 Fig — Highlighted are the bases conserved in all species (black) and respective first amino acids of the proteins (orange). (TIFF) [file ppat.1012788.s004.tiff]

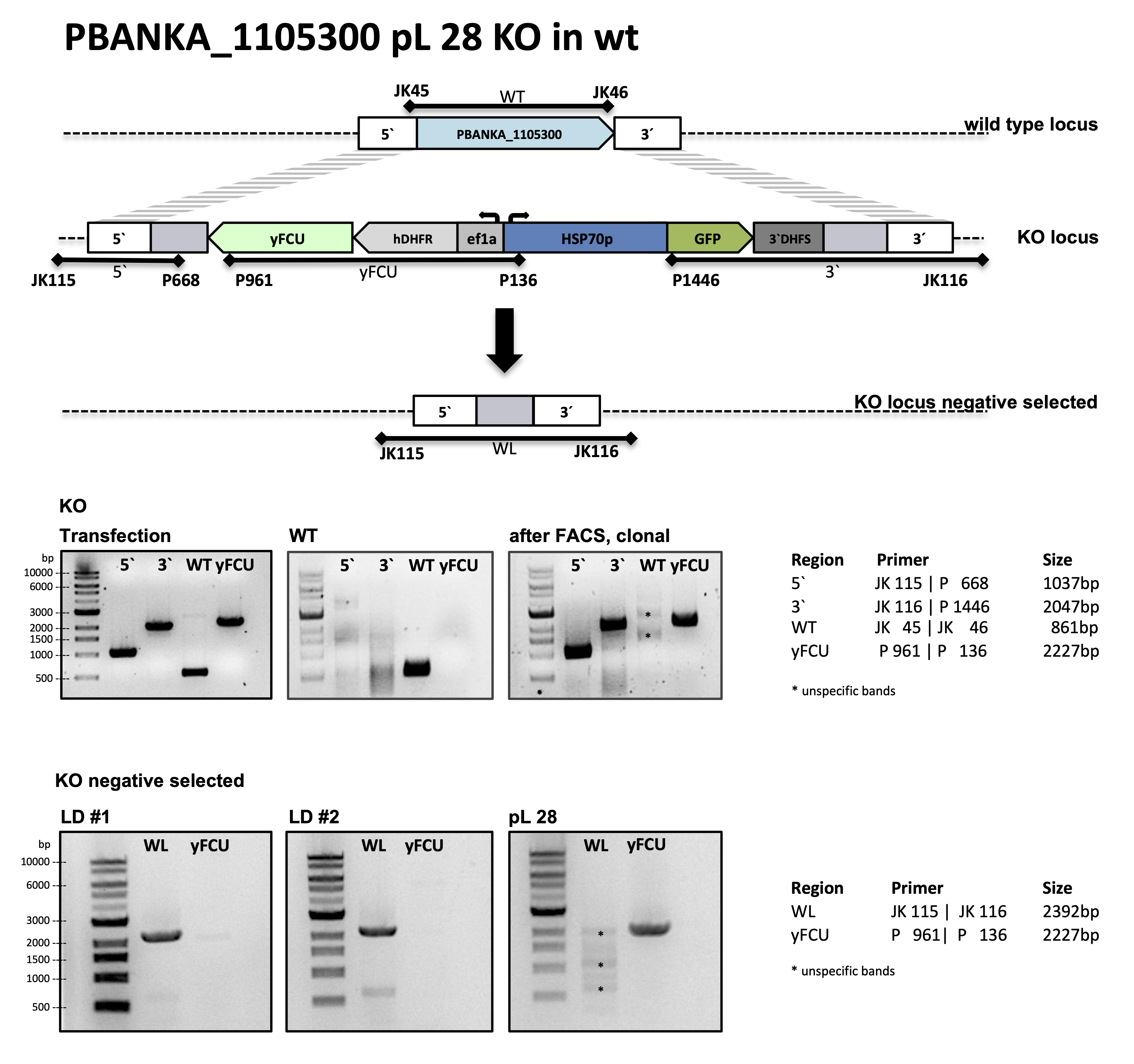

Supplement: S5 Fig — The cartoon shows the cloning strategy and primers used for genotyping. Note that yFCU (Yeast cytosine deaminase-uracil phosphoribosyl transferase fusion protein) was used as a negative selection marker (see methods). (TIFF) [file ppat.1012788.s005.tiff]

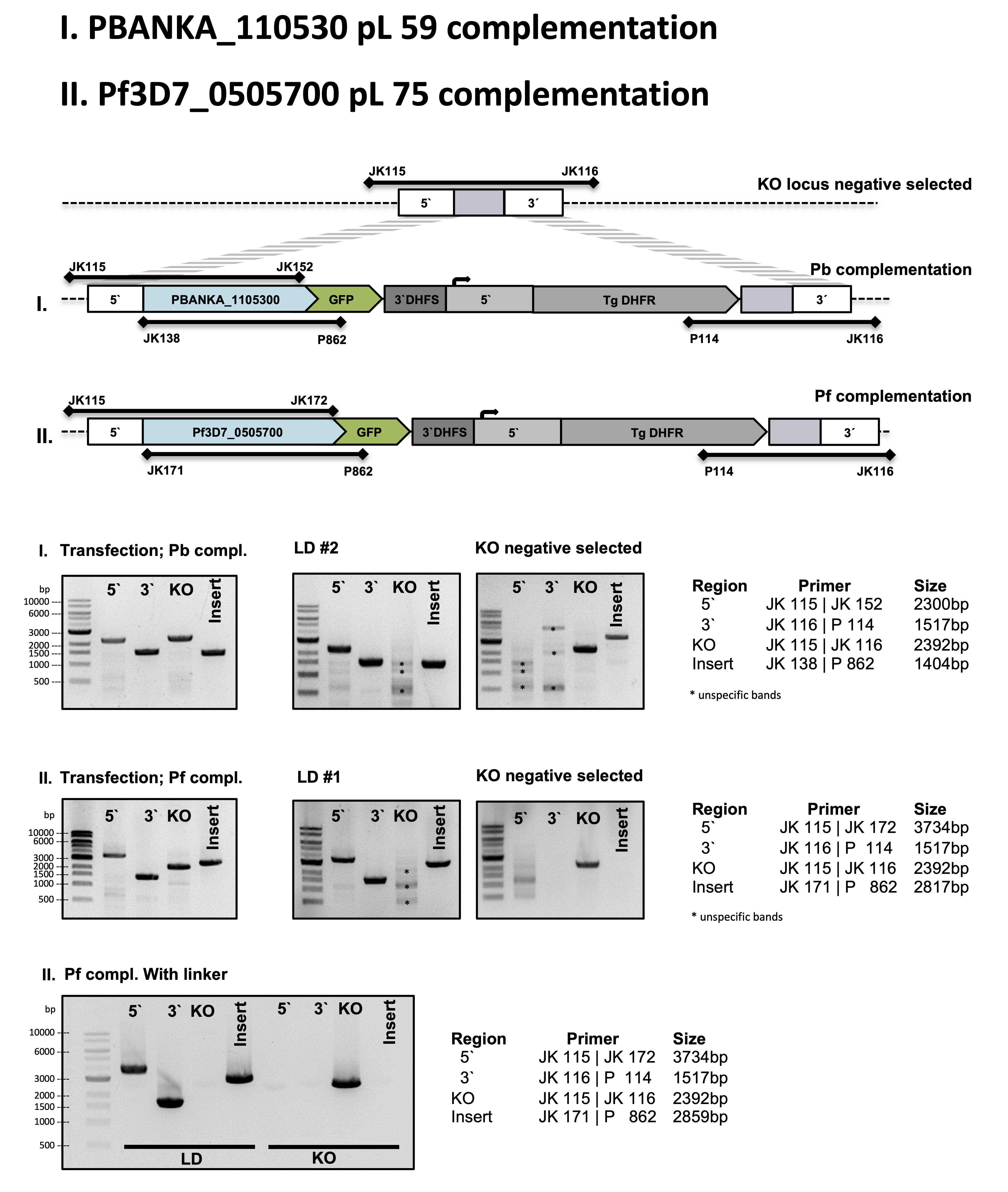

Supplement: S6 Fig — The cartoon shows the cloning strategy and primers used for genotyping. (TIFF) [file ppat.1012788.s006.tiff]

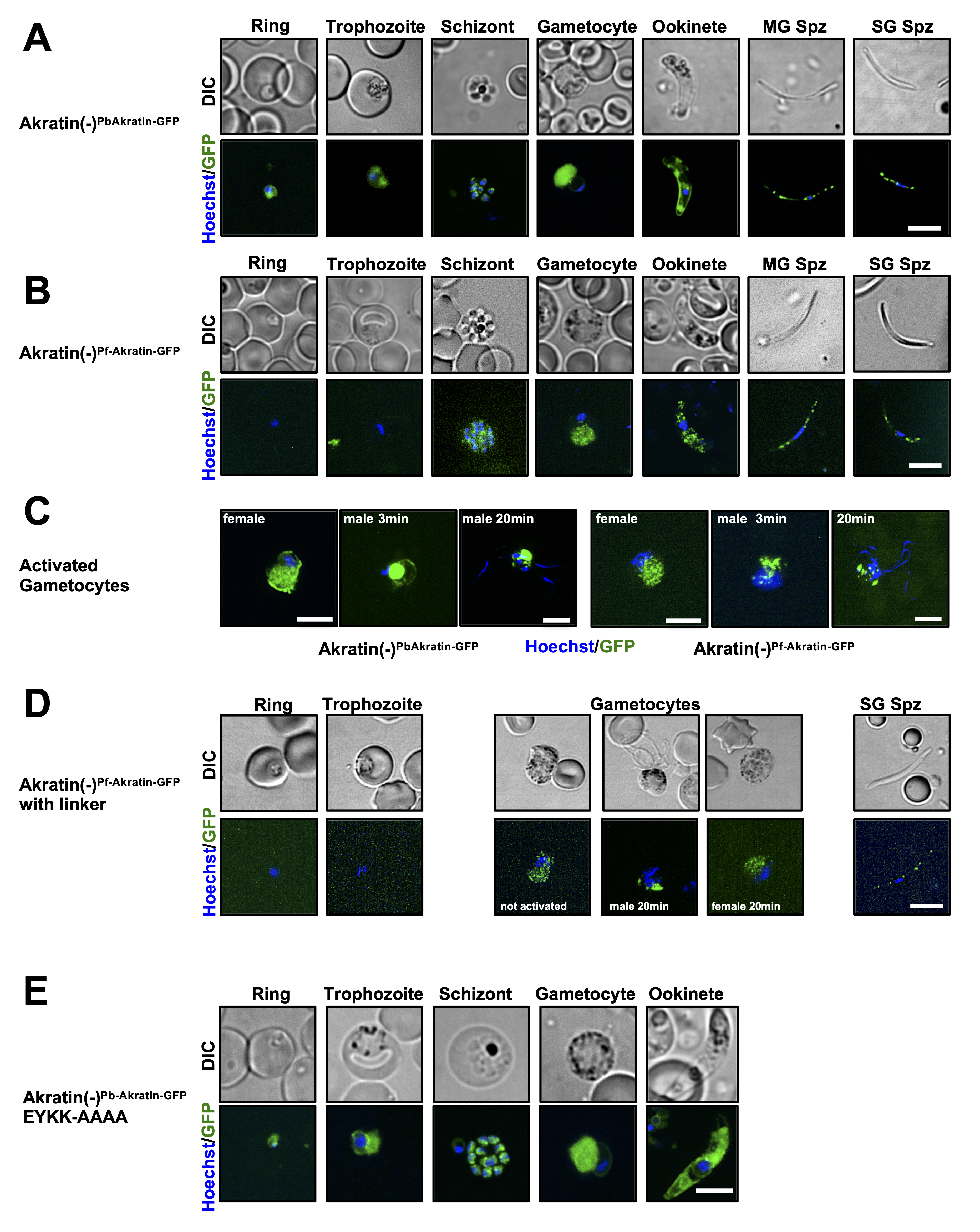

Supplement: S7 Fig — (A) Pbakratin-gfp and (B) Pfakratin-gfp localization in blood and mosquito stages of P. berghei parasites. Nuclei (blue) are stained with Hoechst. DIC: differential interference contrast, MG: midgut, SG: salivary gland. Note the weaker Pfakratin-GFP signal in gametocytes and the difference between the signals in ookinetes. Scale bars: 5 μm. (C) Akratin-GFP localization in activated male and female gametocytes. Scale bars: 5 μm. (D) Pfakratin-GFP with linker. Localization in blood and mosquito stages. Nuclei (blue) are stained with Hoechst. DIC: differential interference contrast, SG: salivary gland. Scale bar: 5μm. (E) Localization of akratin-GFP in the indicated P. berghei life cycle stages. Scale bar: 5 μm. Note that the GFP signal does not correspond to the localization of the intended fusion protein, as GFP is cleaved off. (TIFF) [file ppat.1012788.s007.tiff]

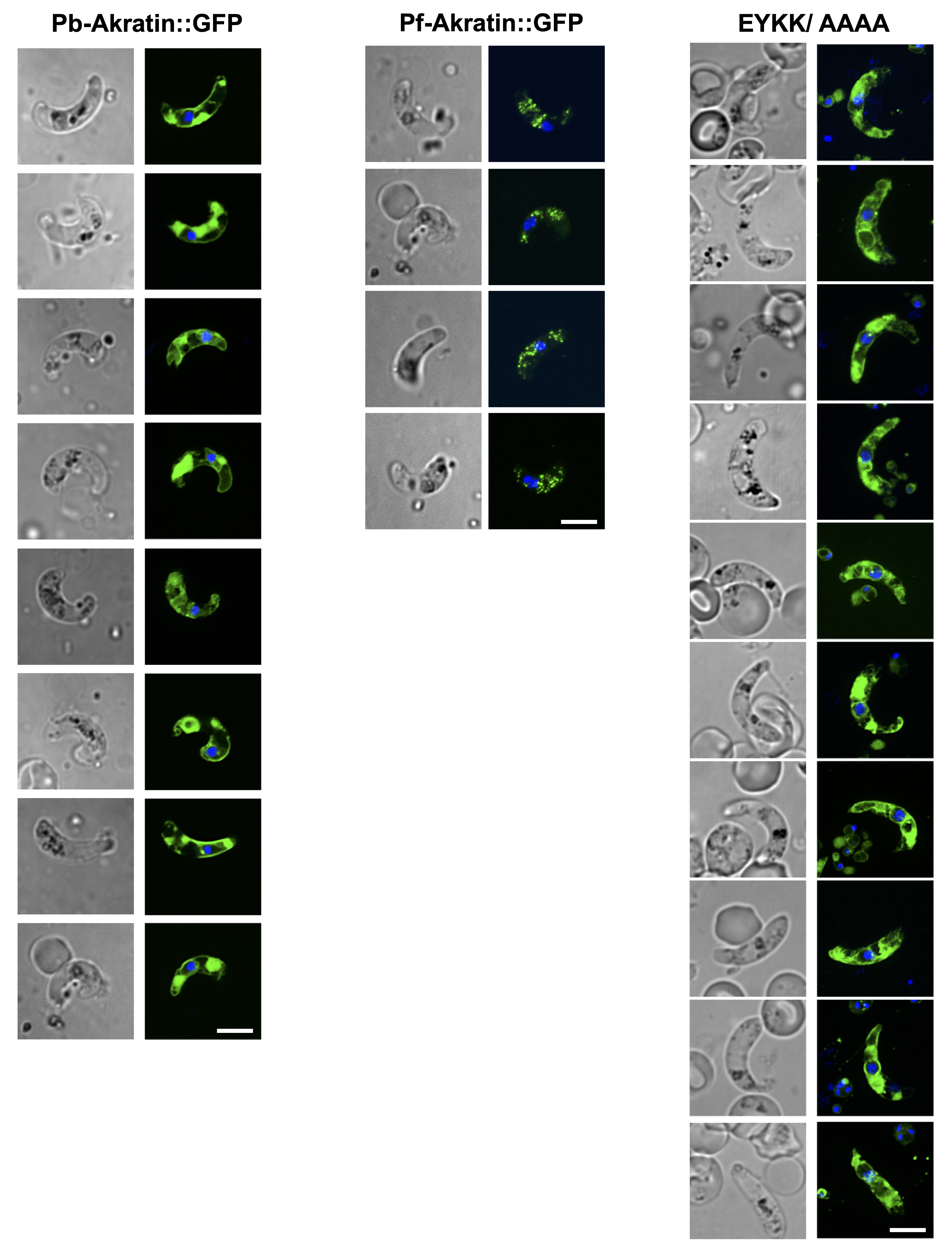

Supplement: S8 Fig — Scale bar: 5 μm. Note that the GFP signal does not correspond to the localization of the intended fusion protein. (TIFF) [file ppat.1012788.s008.tiff]

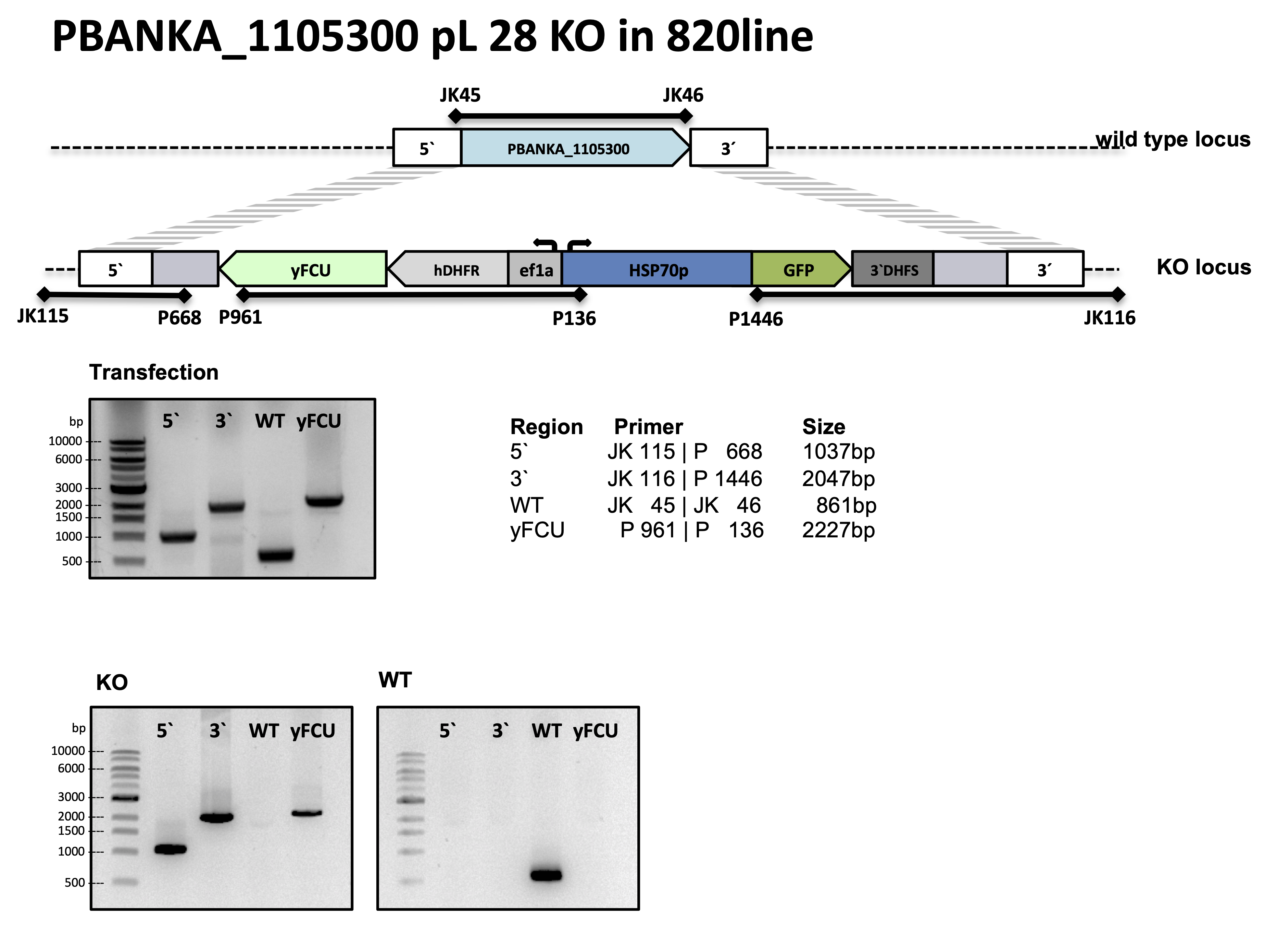

Supplement: S9 Fig — The cartoon shows the cloning strategy and primers used for genotyping. (TIFF) [file ppat.1012788.s009.tiff]

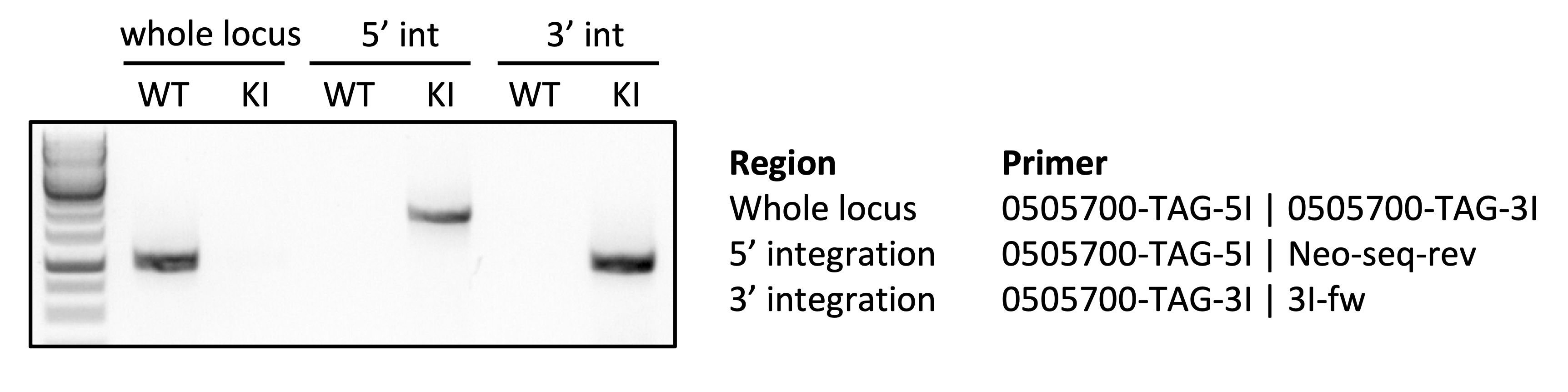

Supplement: S10 Fig — Image shows agarose gel picture of the Integration PCRs. KI = knock-in. Primer combinations are indicated on the right. (TIFF) [file ppat.1012788.s010.tiff]

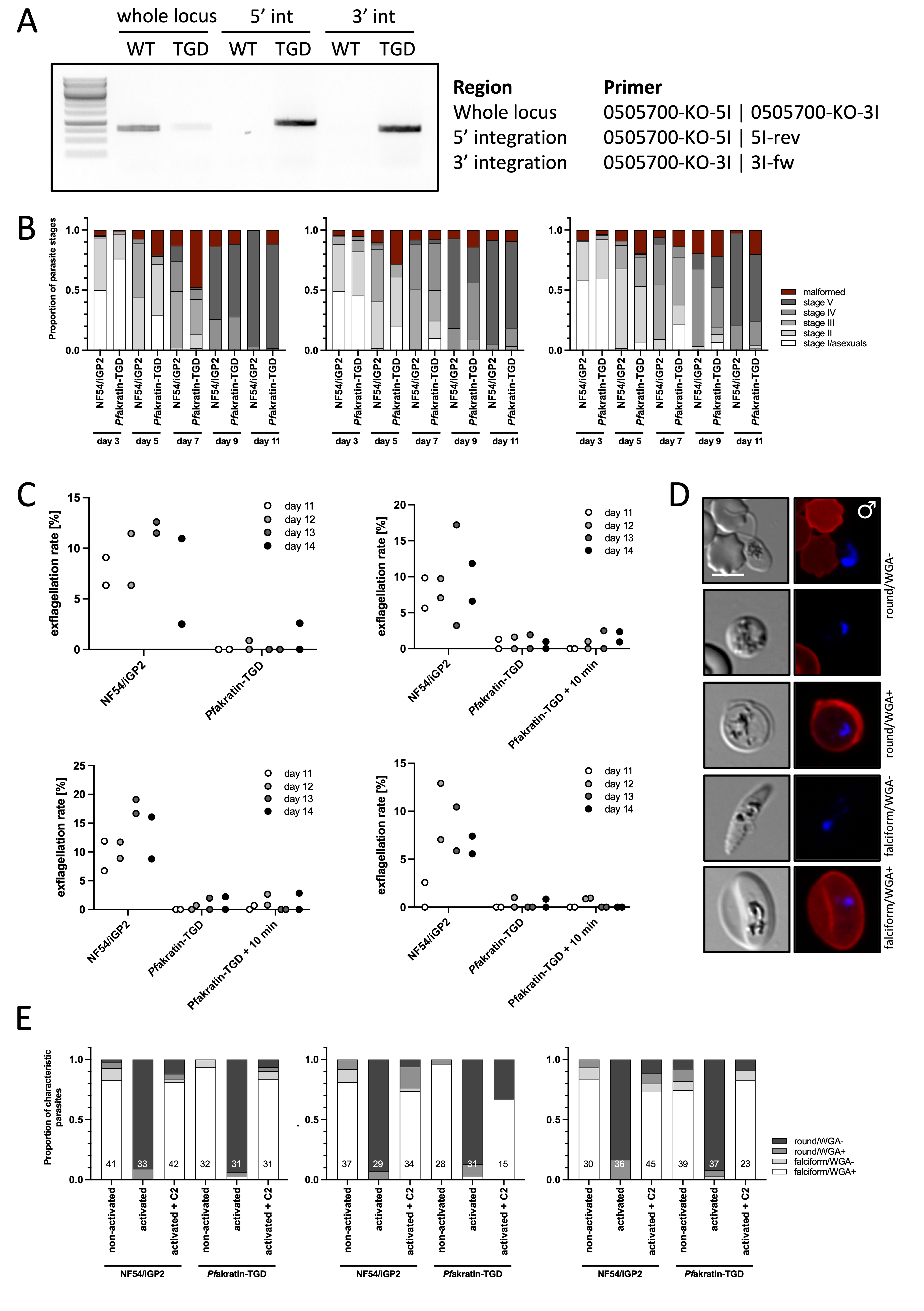

Supplement: S11 Fig — (A) Genotyping of Pfakratin- TGD. Image shows agarose gel picture of the integration PCRs. Primer combinations are indicated on the right. TGD = targeted gene deletion. (B) Gametocyte morphology of three independent experiments. Stages determined by counting them on Giemsa-stained thin blood smears. Normalized to the total number of single-infected RBC. (C) Exflagellation of gametocytes in a 5-min window after 12 min activation in four independent experiments. Extended observation time for Pfakratin-TGD parasites as indicated. Exflagellation was assessed in technical duplicates on four subsequent days as indicated. Dots represent individual data points. (D) Example images for the WGA-based egress assay in (E). Top panels show an exflagellating male gametocyte with flagella and an enlarged nucleus. Scale bar: 5 μm. (E) Egress of stage V gametocytes on day 14 normalized to the total number of gametocytes observed. Counting based on shape and presence/loss of the WGA signal of the RBCM. Numbers within the bars indicate the number of gametocytes counted. (TIFF) [file ppat.1012788.s011.tiff]

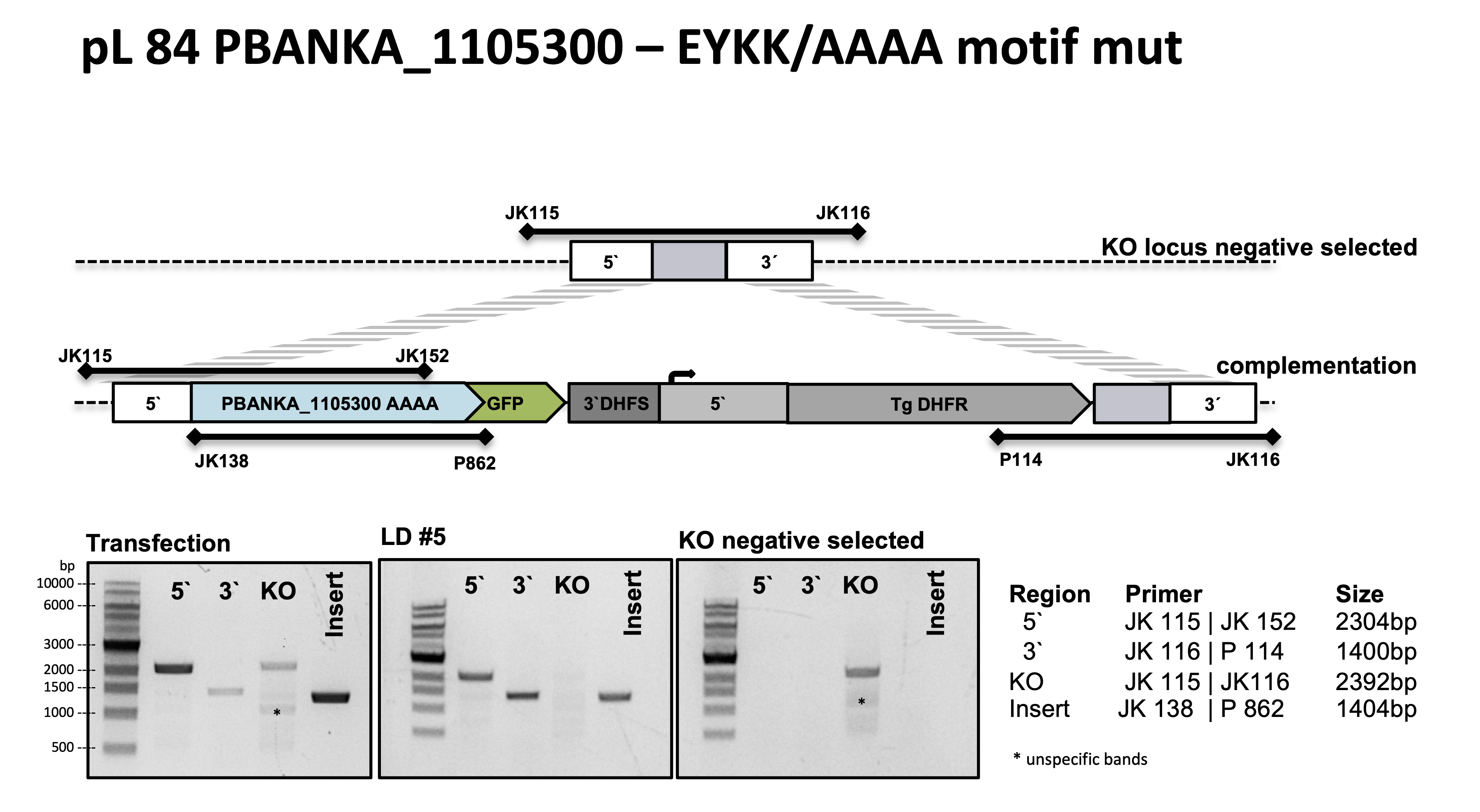

Supplement: S12 Fig — The cartoon shows the cloning strategy and primers used for genotyping. (TIFF) [file ppat.1012788.s012.tiff]

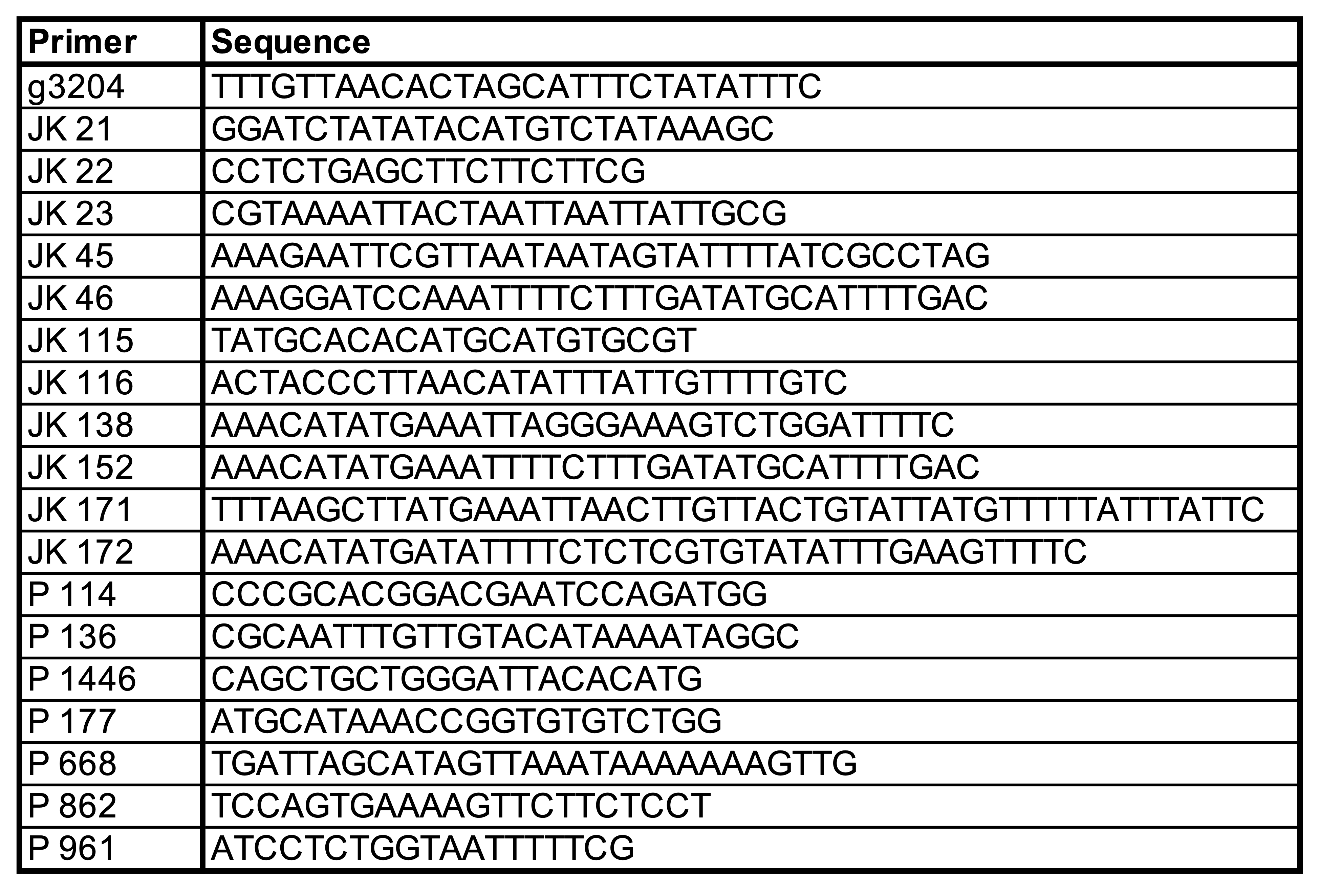

Supplement: S2 Table — (TIFF) [file ppat.1012788.s014.tiff]
