# Supplementary material for: APEX-based proximity labeling in Plasmodium identifies a membrane protein with dual functions during mosquito infection
Source: PLoS Pathog. 2024 Dec 18;20(12):e1012788. doi: 10.1371/journal.ppat.1012788 (PMC11695019; doi:10.1371/journal.ppat.1012788)
Supplement: S1 Table — (PDF) [file ppat.1012788.s013.pdf]

Gene ID		TM	SP	pl8 +H2O2	pl8 -H2O2	wt +H2O2	pl8 +H2O2	pl8 -H2O2	wt +H2O2	SUM pl8+	PlasmoGem phenotype	Lal et al.	function
PBANKA_1300700	LCCL domain-containing protein (CCp1)	0	x	86	0	0	49	0	0	135		x	micronemal
PBANKA_1035200	LCCL domain-containing protein (CCp3)	0	x	71	0	0	33	0	0	104		x	micronemal
PBANKA_0204500	LCCL domain-containing protein (CCp5)	0	x	68	0	0	31	0	0	99		x	micronemal
PBANKA_1437300	endoplasmin, putative	0	x	48	1	0	41	0	0	89	L, essential	x	micronemal
PBANKA_1410300	M1-family alanyl aminopeptidase, putative	0	x	19	0	10	54	0	0	73	L, essential	x	micronemal
PBANKA_0702800	protein disulfide isomerase	0	x	28	0	0	28	0	0	56	L, essential	x	micronemal
PBANKA_1318100	aminopeptidase P, putative (APP)	0	x	6	0	1	27	0	0	33	L, essential	x	micronemal
PBANKA_0830200	high molecular weight rhoptry protein 2	0	x	23	0	0	10	0	0	33		x	micronemal
PBANKA_0914300	protein disulfide isomerase related protein, putative	0	x	19	0	0	10	0	0	29		x	micronemal
PBANKA_1319500	LCCL domain-containing protein CCP2 (CCP2)	0	x	12	0	0	16	0	0	28		x	micronemal
PBANKA_1237800	multidrug resistance protein 1, putative	11		3	0	1	20	0	0	23	L, essential	x	micronemal
PBANKA_1214300	enolase, putative	0		3	0	0	19	0	0	22	L, essential	x	micronemal
PBANKA_1102200	merozoite surface protein 8	1	x	4	0	5	18	0	0	22	L, dispensable	x	micronemal
PBANKA_1034400	plasmepsin IV	1		8	0	7	13	0	0	21	Sig slow	x	micronemal
PBANKA_0209200	parasite-infected erythrocyte surface protein	1	x	14	0	0	6	0	0	20	L, dispensable	x	micronemal
PBANKA_1315300	LCCL domain-containing protein Lap5	0	x	8	0	0	7	0	0	15	L, dispensable		micronemal
PBANKA_0932400	berghepain-2	1		3	0	0	11	0	0	14			micronemal
PBANKA_0942500	thioredoxin-like mero protein, putative	0	x	6	0	0	7	0	0	13	L, essential	x	micronemal
PBANKA_1359700	6-cysteine protein p47	2	x	11	0	0	1	0	0	12	L, dispensable	x	micronemal
PBANKA_0931300	dipeptidyl aminopeptidase 1, putative	1	x	1	0	0	11	0	0	12	L, essential	x	micronemal
PBANKA_0826700	parasitophorous vacuolar protein 5	0	x	3	0	3	9	0	0	12		x	micronemal
PBANKA_0416000	high molecular weight rhoptry protein 3, putative	0	x	6	0	0	4	0	0	10	L, essential	x	micronemal
PBANKA_0800500	chitinase	0	x	0	0	0	9	0	0	9	Sig slow	x	micronemal
PBANKA_1137000	bergheilysin	0		2	0	0	6	0	0	8	L, essential	x	micronemal
PBANKA_1113400	secreted ookinete protein	0	x	7	0	0	1	0	0	8		x	micronemal
PBANKA_0304900	serine repeat antigen 3	0		2	0	0	6	0	0	8			micronemal
PBANKA_1321700	berghepain-1	1		6	0	0	1	0	0	7	Sig slow	x	micronemal
PBANKA_0703900	receptor for activated c kinase, putative	0		2	0	0	5	0	0	7		x	micronemal
PBANKA_0207000	calcium-transporting ATPase, putative	8		6	0	0	0	0	0	6	L, essential	x	micronemal
PBANKA_0407300	Ookinete motiliy deficient protein (OMD)	0	x	4	0	0	2	0	0	6	L, dispensable		micronemal
PBANKA_1224500	Dpy-19-like C-mannosyltransferase, putative	11	x	6	0	0	0	0	0	6			micronemal
PBANKA_0514900	ookinete surface protein P28	1	x	2	0	0	3	0	0	5	Sig slow	x	micronemal
PBANKA_1229000	Plasmodium exported protein (PHIST), unknown function	1		0	0	0	5	0	0	5			micronemal
PBANKA_0305000	serine repeat antigen 2	0	x	1	0	0	4	0	0	5	L, dispensable		micronemal
PBANKA_0412900	circumsporozoite- and TRAP-related protein	2	x	0	0	0	4	0	0	4		x	micronemal
PBANKA_0911400	CLPTM1 domain-containing protein, putative	6		3	0	0	1	0	0	4	L, dispensable	x	micronemal
PBANKA_0819000	glideosome-associated protein 50, putative	2	x	2	0	0	2	0	0	4		x	micronemal
PBANKA_1037800	secreted ookinete adhesive protein	0	x	1	0	0	3	0	0	4	L, dispensable	x	micronemal
PBANKA_1228900	von Willebrand factor A domain-related protein	0	x	0	0	0	4	0	0	4		x	micronemal
PBANKA_1338700	plasmepsin V, putative	1	x	3	0	0	1	0	0	4	L, essential		micronemal
PBANKA_1434400	secreted ookinete protein, putative	0	x	3	0	0	1	0	0	4	L, dispensable		micronemal
PBANKA_1101400	rhoptry-associated protein 2/3	0	x	0	0	0	3	0	0	3	Sig slow	x	micronemal
PBANKA_1309900	M17 leucyl aminopeptidase, putative	0		1	0	0	2	0	0	3	Sig slow		micronemal
PBANKA_0919100	parasitophorous vacuolar protein 1	0	x	0	0	0	3	0	0	3	Sig slow		micronemal
PBANKA_0809400	plasma membrane protein 1	2	x	3	0	0	0	0	0	3	L, dispensable		micronemal
PBANKA_0623100	tryptophan-rich protein	1		0	0	0	3	0	0	3			micronemal
PBANKA_1107600	6-cysteine protein	2	x	0	0	0	2	0	0	2	L, dispensable	x	micronemal
PBANKA_0915000	apical membrane antigen 1	1	x	0	0	0	2	0	0	2		x	micronemal
PBANKA_0712200	BEM46-like protein, putative	3	x	0	0	0	2	0	0	2	L, dispensable	x	micronemal
PBANKA_1145800	membrane associated histidine-rich protein 1a	1		1	0	2	1	0	0	2		x	micronemal
PBANKA_0515000	ookinete surface protein P25	2	x	0	0	0	2	0	0	2	L, dispensable	x	micronemal
PBANKA_1008500	translocon component PTEX150	0	x	0	0	0	2	0	0	2	L, essential	x	micronemal
PBANKA_0408500	parasite-infected erythrocyte surface protein PIESP	0	x	2	0	0	0	0	0	2			micronemal
PBANKA_1335800	protein disulfide-isomerase, putative	0	x	2	0	0	0	0	0	2	L, dispensable		micronemal
PBANKA_0619200	secreted ookinete protein, PSOP1	0	x	0	0	0	2	0	0	2	L, dispensable		micronemal
PBANKA_0522500	transmembrane emp24 domain-containing protein, putative	2	x	2	0	0	0	0	0	2			micronemal
**PBANKA_1105300**	**conserved Plasmodium protein, unknown function**	**4**	**x**	**13**	**0**	**0**	**3**	**0**	**0**	**16**			**unknown**
PBANKA_1243600	conserved Plasmodium protein, unknown function	2	x	1	0	0	4	0	0	5	L, essential		unknown
PBANKA_0620700	conserved Plasmodium protein, unknown function	0	x	4	0	0	1	0	0	5			unknown
PBANKA_0403800	conserved Plasmodium membrane protein, unknown function	12		0	0	0	3	0	0	3			unknown
PBANKA_0806400	conserved protein, unknown function	2	x	3	0	0	0	0	0	3	L, dispensable		unknown
PBANKA_1454200	conserved Plasmodium protein, unknown function	0		2	0	0	0	0	0	2	L, essential		unknown
PBANKA_0818900	endoplasmic reticulum chaperone BiP, putative	0	x	46	1	2	40	0	1	86			Chaperone
PBANKA_1357200	heat shock protein 110, putative	0	x	21	0	0	24	0	0	45	L, essential	x	Chaperone
PBANKA_0711900	heat shock protein 70	0		13	0	4	26	1	1	39	L, essential	x	Chaperone
PBANKA_0805700	heat shock protein 90, putative	0		4	1	1	20	0	0	24	Sig slow	x	Chaperone
PBANKA_1127800	DnaJ protein, putative	0	x	9	0	0	1	0	0	10	Sig slow	x	Chaperone
PBANKA_0938300	heat shock protein J2, putative	1	x	9	0	0	1	0	0	10	L, essential	x	Chaperone
PBANKA_0706800	DnaJ protein, putative	3		7	0	0	1	0	0	8	Sig slow	x	Chaperone
PBANKA_1219300	heat shock protein 110, putative	0		0	0	0	7	0	0	7			Chaperone
PBANKA_0310900	T-complex protein 1 subunit theta, putative	0		0	0	0	4	0	0	4			Chaperone
PBANKA_0820000	DnaJ protein, putative	6		3	0	0	0	0	0	3	L, essential	x	Chaperone
PBANKA_0916200	T-complex protein 1 subunit alpha, putative	0		0	0	0	3	0	0	3		x	Chaperone
PBANKA_0405200	T-complex protein 1 subunit beta, putative	0		0	0	0	3	0	0	3	L, essential	x	Chaperone
PBANKA_0406500	T-complex protein 1 subunit eta, putative	0		0	0	0	3	0	0	3	L, essential	x	Chaperone
PBANKA_0929900	heat shock protein 90, putative	0		1	0	0	2	0	0	3			Chaperone
PBANKA_1463900	HSP20-like chaperone, putative	0	x	2	0	0	1	0	0	3			Chaperone
PBANKA_1444100	T-complex protein 1 subunit gamma, putative	0		0	0	0	3	0	0	3	L, essential		Chaperone

PBANKA_1224200	DnaJ protein, putative	0		**0**	0	0	**2**	0	0	**2**			Chaperone
PBANKA_1134100	T-complex protein 1 subunit delta, putative	0		**0**	0	0	**2**	0	0	**2**	L, essential		Chaperone
PBANKA_1218200	T-complex protein 1 subunit epsilon, putative	0		**0**	0	0	**2**	0	0	**2**	L, essential		Chaperone
PBANKA_0522700	alpha tubulin 2	0		**9**	0	0	**12**	0	0	**21**			contaminant
PBANKA_1206900	tubulin beta chain, putative	0		**3**	0	1	**14**	0	0	**17**	L, essential	x	contaminant
PBANKA_0938400	endoplasmic reticulum-resident calcium binding protein	0	x	**9**	0	0	**7**	0	0	**16**		x	contaminant
PBANKA_1030600	p1/s1 nuclease, putative	0	x	**3**	0	3	**13**	0	0	**16**		x	contaminant
PBANKA_1459300	actin I	0		**3**	0	1	**12**	0	0	**15**	L, essential	x	contaminant
PBANKA_1326400	glyceraldehyde-3-phosphate dehydrogenase	0		**2**	0	1	**12**	0	0	**14**	L, essential	x	contaminant
PBANKA_1337400	nucleoside-diphosphatase, putative	1	x	**9**	0	0	**4**	0	0	**13**	L, essential	x	contaminant
PBANKA_1125600	pyruvate kinase, putative	0		**3**	0	0	**10**	0	0	**13**	L, essential	x	contaminant
PBANKA_1133300	elongation factor 1-alpha	0		**4**	0	0	**9**	0	0	**13**			contaminant
PBANKA_1315500	sortilin, putative	2	x	**9**	0	0	**3**	0	0	**12**		x	contaminant
PBANKA_1314800	elongation factor 2, putative	0		**0**	0	0	**12**	0	0	**12**	L, essential	x	contaminant
PBANKA_1365500	exported protein IBIS1	1		**1**	0	1	**9**	0	0	**10**		x	contaminant
PBANKA_1429300	integral membrane protein GPR180, putative	8	x	**8**	0	0	**2**	0	0	**10**	L, dispensable		contaminant
PBANKA_1217700	ATP-dependent RNA helicase DDX6	0		**0**	0	0	**9**	0	0	**9**	L, dispensable	x	contaminant
PBANKA_1329300	40S ribosomal protein S3, putative	0		**3**	0	0	**5**	0	0	**8**		x	contaminant
PBANKA_1308600	fructose-bisphosphate aldolase 2	0		**0**	0	0	**8**	0	0	**8**	L, essential	x	contaminant
PBANKA_1365200	thioredoxin-related protein, putative	1	x	**5**	0	0	**3**	0	0	**8**		x	contaminant
PBANKA_0924300	endoplasmic reticulum oxidoreductin, putative	2	x	**7**	0	0	**1**	0	0	**8**			contaminant
PBANKA_1426300	ER membrane protein complex subunit 1, putative	3	x	**7**	0	0	**0**	0	0	**7**	L, essential	x	contaminant
PBANKA_1122900	hexokinase, putative	0		**1**	0	0	**6**	0	0	**7**	L, essential		contaminant
PBANKA_1302800	thioredoxin peroxidase 1	0		**2**	0	0	**4**	0	0	**6**	L, dispensable	x	contaminant
PBANKA_0712600	14-3-3 protein	0		**1**	0	0	**4**	1	0	**5**	L, essential	x	contaminant
PBANKA_1450300	ATP synthase subunit beta, mitochondrial	0		**1**	0	0	**4**	0	0	**5**	Sig slow	x	contaminant
PBANKA_1118600	cell division cycle protein 48 homologue, putative	0		**0**	0	0	**5**	0	0	**5**	L, essential	x	contaminant
PBANKA_1331900	eukaryotic initiation factor 4a, putative	0		**2**	0	1	**3**	0	0	**5**		x	contaminant
PBANKA_0930300	GTP-binding nuclear protein RAN/TC4, putative	0		**0**	0	0	**5**	0	0	**5**	L, essential	x	contaminant
PBANKA_1231800	60S ribosomal protein L12, putative	0		**2**	0	1	**3**	0	0	**5**	L, essential		contaminant
PBANKA_0520200	ADP/ATP transporter on adenylate translocase, putative	3		**3**	0	1	**2**	0	0	**5**	Sig slow		contaminant
PBANKA_1418300	golgi protein 1, putative	0	x	**5**	0	0	**0**	0	0	**5**			contaminant
PBANKA_1032700	CUGBP Elav-like family member 2, putative	0		**0**	0	0	**4**	0	0	**4**	L, dispensable	x	contaminant
PBANKA_1238800	karyopherin beta, putative	0		**0**	0	0	**4**	0	0	**4**	L, dispensable	x	contaminant
PBANKA_1141700	ubiquitin-60S ribosomal protein L40, putative	0		**2**	0	1	**2**	0	0	**4**		x	contaminant
PBANKA_0619100	40S ribosomal protein S5, putative	0		**1**	0	0	**3**	0	0	**4**			contaminant
PBANKA_1317500	glucose-6-phosphate dehydrogenase-6-phosphogluconolactonase	0		**0**	0	0	**4**	0	0	**4**	Sig slow		contaminant
PBANKA_0104700	long chain polyunsaturated fatty acid elongation enzyme	7		**2**	0	0	**2**	0	0	**4**	L, dispensable		contaminant
PBANKA_1326000	GTP-binding protein, putative	3	x	**3**	0	0	**0**	0	0	**3**		x	contaminant
PBANKA_1301300	trailer hitch homolog CITH	0		**0**	0	0	**3**	0	0	**3**		x	contaminant
PBANKA_1201900	40S ribosomal protein S20e, putative	0		**0**	0	0	**3**	0	0	**3**			contaminant
PBANKA_0505100	ADP-ribosylation factor, putative	0		**1**	0	0	**2**	0	0	**3**			contaminant
PBANKA_1426900	ATP-dependent RNA helicase DBP1, putative	0		**0**	0	0	**3**	0	0	**3**	Sig slow		contaminant
PBANKA_1003800	V-type proton ATPase subunit B, putative	0		**0**	0	0	**3**	0	0	**3**			contaminant
PBANKA_0924800	golgi protein 2	1	x	**2**	0	0	**0**	0	0	**2**		x	contaminant
PBANKA_0522800	40S ribosomal protein S19, putative	0		**0**	0	0	**2**	0	0	**2**		x	contaminant
PBANKA_1364200	60S ribosomal protein L17, putative	0		**0**	0	0	**2**	0	0	**2**		x	contaminant
PBANKA_0417700	alpha tubulin 1	0		**0**	0	0	**2**	0	0	**2**	L, dispensable	x	contaminant
PBANKA_0306800	ATP-dependent RNA helicase UAP56, putative	0		**0**	0	0	**2**	0	0	**2**	L, essential	x	contaminant
PBANKA_1406700	carbamoyl phosphate synthetase, putative	0		**0**	0	0	**2**	0	0	**2**	L, essential	x	contaminant
PBANKA_1423300	DNA/RNA-binding protein Alba 1, putative	0		**0**	0	0	**2**	0	0	**2**		x	contaminant
PBANKA_0941800	histone H2B, putative	0		**0**	0	0	**2**	0	0	**2**	L, essential	x	contaminant
PBANKA_1135000	YOP1-like protein, putative	3	x	**0**	0	0	**2**	0	0	**2**		x	contaminant
PBANKA_0510900	40S ribosomal protein S2, putative	0		**0**	0	0	**2**	0	0	**2**	L, essential		contaminant
PBANKA_1202400	60S ribosomal protein L13, putative	0		**0**	0	0	**2**	0	0	**2**	L, essential		contaminant
PBANKA_0407600	asparagine synthetase [glutamine-hydrolyzing]	0		**0**	0	0	**2**	0	0	**2**	Sig slow		contaminant
PBANKA_0919900	ATP-dependent 6-phosphofructokinase, putative	0		**0**	0	0	**2**	0	0	**2**	L, dispensable		contaminant
PBANKA_0816400	ATP-dependent 6-phosphofructokinase, putative	0		**0**	0	0	**2**	0	0	**2**			contaminant
PBANKA_0819100	cytochrome b5, putative	1	x	**0**	0	0	**2**	0	0	**2**	Sig slow		contaminant
PBANKA_0505800	deoxyribose-phosphate aldolase, putative	0		**0**	0	0	**2**	0	0	**2**			contaminant
PBANKA_1364300	glycerol kinase, putative	0		**0**	0	0	**2**	0	0	**2**			contaminant
PBANKA_1220200	lysophospholipase, putative	0		**0**	0	0	**2**	0	0	**2**			contaminant
PBANKA_1210900	phosphoglucomutase, putative	0		**0**	0	0	**2**	0	0	**2**	L, dispensable		contaminant
PBANKA_1223300	ras-related protein Rab-18, putative	0		**0**	0	0	**2**	0	0	**2**			contaminant
PBANKA_0611600	ribonucleoside-diphosphate reductase large subunit, putative	0		**0**	0	0	**2**	0	0	**2**	L, essential		contaminant
