# Supplementary material for: APEX-based proximity labeling in Plasmodium identifies a membrane protein with dual functions during mosquito infection
Source: PLoS Pathog. 2024 Dec 18;20(12):e1012788. doi: 10.1371/journal.ppat.1012788 (PMC11695019; doi:10.1371/journal.ppat.1012788)
Supplement: Attachment — Submitted filename: 110982_1_rebuttal_3136765_sjc6g9.docx [file ppat.1012788.s016.docx]

Dear editors,

we thank the reviewers for their constructive and generally positive comments. Please find our answers to the specific questions below in blue. A kex and completely justified critique was that we did not show if the akratin-GFP fusion protein was indeed a fusion protein by Wester blotting. To address this, we first did Western blots and found it was not a fusion protein. We then generated another parasite with a different linker following previous work that showed similar problems. However, this also failed. We therefore turned to *P. falciparum*, where we readily obtained an akratin-mScalet expressing parasite line. Western blotting showed that the fusion protein was intact, while in *P. berghei* all fusion protein attempts let to cleaved GFP. We discuss this in the paper. These data are presented in a new figure and were generated by collaborators who are now listed as authors. We hope that with the included extra data you find this paper now in shape for publication.

Sincerely

Jessica Kehrer and Freddy Frischknecht

Reviewer #1 (Evidence, reproducibility and clarity (Required)): 

Proximity labelling using BirA* has emerged as a highly successful approach to identify interaction partners and proteins in compartments of a protein of interest in the living cell. A number of recent studies applied this approach with malaria parasites and demonstrated its usefulness. However, a drawback of using BirA* is the time required to obtain good yields of biotinylated proteins (usually many hours). APEX is a much faster alternative to BirA* that however has so far not been used in apicomplexan parasites. Here Kehrer and colleagues use APEX2 to identify proteins of the micronemes of mature Ookinetes, a task that due to the short time available for labelling, would have been difficult with BirA*. From the obtained hits the authors chose a protein they named akratin and carried out a detailed functional analysis. They found that akratin is needed for microgametogenesis and for ookinete migration. Complementation with either the Pb or Pf GFP-tagged version of akratin rescued all defects of the mutant, despite the low sequence similarity and differing number of predicted transmembrane segments in the P. falciparum protein. Interestingly a mutant form of this protein with an ablated putative trafficking motif in its C-terminus also caused an ookinete migration phenotype but microgametogenesis was unaffected, hinting at intricacies in its function. 

This is a clearly written and interesting paper reporting the first use of APEX-based BioID in malaria parasites and demonstrates that it is possible to take advantage of BioID in short lived stages. The ookinete microneme proteome reported here compares favourably with that derived from organelle purifications and will provide a resource to identify the proteins and understand their involvement in migration and adhesion of this and possibly other parasite stages. The example protein chosen for functional analysis in this work nicely illustrates this. A validation of some more of the unknown hits to be true microneme proteins would have been beneficial, but given the high number of known true positives in the hit list this is not absolutely essential for the paper. Overall this manuscript therefore provides a nice piece of work adding a list of proteins for future study and a new player important for mosquito infection.

Thanks for this overall very positive assessment.

**Minor points:** 

1.several proteins taken as true hits in TableS1 are not obvious to me, such as plasmepsin V and several exported proteins. Are they expected to be trafficked via the micronemes in ookinetes?

We hope not to give the impression that we found “true hits”. We indeed label them as “potential micronemal” (line 174). Compared to motile merozoites and sporozoites, ookinetes do not have rhoptries, yet do rely on proteases for epithelial cell traversal in the midgut. Hence, we didn’t want to exclude the possibility of certain proteins such as proteases or putative rhoptry proteins to localize to micronemes.

2.Fig. 1C: '+ Streptavidin', this should be '+ streptavidin beads'

Thanks for pointing this out. We have changed it accordingly.

3.please insert a referral to Figure S2 (similarity of the Plasmodium akratin homologs) somewhere around line 180, the only reference to this figure I could find in the text was in line 214. Figure S3 (line 184) is mentioned in a context to support the absence of homologues outside Plasmodium spp but shows the generation of the akratin KO. Maybe this should have been a citation to Figure S2, but then something is missing from that figure.

Thanks. We now revised the entire manuscript carefully paying attention to figures and numbering.

4.line 192, it might be useful to clarify in the text what 10 and 7 blood stage growth means (multiplication rate?).

The values refer to the multiplication rate of blood- stage parasites within 24h. We have added the additional information to make this clearer. The text now reads (line 211-213):

“During blood-stage development, the resulting polyclonal parasites lacking PBANKA_1105300 exhibited a significantly reduced multiplication rate within 24 hours, decreasing from around 10 in wild-type parasites to seven”

5.the observation that akratin as a multi TM protein with signal peptide seems to be soluble in the parasite is rather unlikely. Is there a precedent for this? My best guess would be that this is GFP alone due to degradation or processing of the akratin-GFP fusion. A Western blot, if sufficient material is available, would clarify this. In regards to the localisation of akratin in the different stages it should also be taken into account that calling anything 'vesicular' based on fluorescent microscopy is rather speculative.

We have performed western blot analyses and indeed we could only detect a band at the size of GFP suggesting that indeed the fusion protein is cleaved, likely in the ER, hence giving the vesicular staining. We observed a similar problem with profilin-GFP (Moreau et al. Plos Path 2016), which could be overcome by using a different linker. Therefore, we transfected parasites with a new construct containing a longer linker. Yet, again this yielded only free GFP.

75kd

25kd

Exemplary WB of Pb Akratin- GFP

We then asked our colleagues in Hamburg (now co-authors on the paper) to generate a *P. falciparum* line with fluorescently (mScarlet) tagged *P. falciparum* akratin, which surprisingly worked just fine. Here the protein appears to be membrane associated (endomembrane system, most likely ER) corresponding well with the transmembrane domain prediction. Therefore, we have now rewritten the respective parts. The new data on *P. falciparum* is now included in the new Figure 4, while images of *P. berghei* parasites are moved into the supplements.

6.line 199, how can the low number of ookinetes affect their speed? Could this remark have been intended for the next sentence (199-201/Fig. 2I) as the lack of transmission to mosquitoes may have been due to the reduced number of ookinetes rather than a deficiency of individual ookinetes? To exclude the latter, were parasite number used matched to exclude that this was the case in Fig. 2I?

Sorry for the misunderstanding we have changed this section.

7.line 272-4, is there a way to quantify the phenotype in ookinetes, e.g. using intensity profile plots showing the distribution across the cell? Many cells still seem to show peripheral staining in the trafficking mutant. Could it again be that some of the protein is processed and the increased cytoplasmic staining represents GFP alone (see also comment 5)? In that case the level of processing rather than trafficking (alone) might be affected in the mutant.

Please see our answer to point 5.

8.lines 396 and 397, there is a minuscule -1 before the chemicals in my pdf.

Thanks. We have removed this from the text.

9.line 58. remove 'not' and 'or': neither been identified nor been characterized

We have corrected this in the text. The sentence now reads (line 58/59):

“To this end proteins on the parasite surface can in principle be targeted by antibodies or drugs; yet many of these proteins have neither been identified nor been characterized in depth.” 

10.lines 88 to 94: English could be polished some more in this part. Line 91, replace 'ones', e.g. with 'proteins'

We now reworked the text and hope to have improved it.

11.lines 245 and 253: add commas after (Figure 4B) and (Figure 4D), respectively

Commas are now added at the respective positions.

12.line 254; change possibly into possible

We have corrected this in the text.  

13.line 296-71: it might be helpful to mention in the text that this was again the complementation strategy (used for the Pb and Pf akratin)

We have added this information to make it clearer.

14.while going beyond the scope of this manuscript, would it be possible to use the APEX introduced here to label structures in EM? A comment on this might be useful for readers interested in using this domain.

We didn’t test using Apex for EM labelling or live cell imaging ourself. Yet in a recent publication (Xu *et al*. Nat Comm 2024) it has been successfully used to label de-ubiquitinase (or ubiquitin carboxyl-terminal hydrolase 1, UBP1) for high resolution EM in *Plasmodium*.

Reviewer #1 (Significance (Required)): 

This is the first use of APEX2 in apicomplexans and this will be very useful for the field. The ookinete microneme proteome will provide a resource to study key aspects of this stage. The unknown proteins in the hit list still have to be confirmed to be true positives, but given the high number of true positives among the known proteins, the success rate should be acceptable. Akratin is a new protein among several known to be important for transmission to the mosquito host. As illustrated by this and other studies, many important questions remain how microgametes egress from the cell and how in vitro gliding and mosquito gut wall penetration relate. Akratin, together with the previously known proteins, will be instrumental in solving these questions. 

My expertise (to put this assessment into perspective): I am a cell biologist working with P. falciparum blood stages and have no or limited experience with mosquito stages and P. berghei. My grasp of the technical difficulties to work with ookinetes and other mosquito stages as presented in this manuscript are therefore limited. Nevertheless, it appears to me that the work is well done and that the overall outcome from this paper is significant and will be of great interest to the field. 

**Referees Cross-Commenting** 

I think the other reviewer's request to carry out a co-loc experiment with a microneme marker to show that akratin and SOAP-APEX are in the correct location is very reasonable and should be done. I also share the view about the trafficking domain. This is reflected in my comment (points 5 and 7) on quantifying the phenotype of the 'trafficking mutant' and the possbility of not looking at the full-length protein as it may be a processed/degraded version of the protein that still contains GFP. Western blots, if possible with these parasite stages, might clear this up. 


Reviewer #2 (Evidence, reproducibility and clarity (Required)): 

In this manuscript by Kehrer et al., use an elegant Apex2 BioID method to identify novel putative microneme proteins by mass-spectrometry and pick one candidate for further characterization. They identify a novel putative microneme protein they name Akratin which they characterize through targeted gene deletion and a series of complementation experiments. This reveals first that akratin appears to be functioning in male gamete egress, and though complementation using a putative trafficking mutant, also in midgut traversal. 

Overall the study is thoroughly performed but some of the conclusions are not fully supported. 

1)The newly identified microneme protein is still putative in my mind as the authors have not co-localized it with another marker. This is crucial for conclusions about its putative function and crucial for the trafficking experiment as explained below. It is also important given the high number of putative false positives in the BioID experiment.

We totally agree, yet as mentioned in answer to reviewer 1 Q5 and to Q3 below we failed to generate a fusion protein in *P. berghei*. However, we did manage now to generate a *P. falciparum* NF54 line expressing akratin-mScarlet. In the earlier stages, the localization is indeed more reminiscent of ER than the secretory organelles. However repeated attempts to generate ookinetes were unsuccessful. We thus rephrased our conclusions throughout the manuscript.

2)I would consider it essential to also localise the Apex2 tagged SOAP protein as the authors cannot be sure that there is a partial mislocalisation of the protein leading to false positives.

We tried to do so by using the anti-myc antibody and our parasite line expressing SOAP-Apex-myc. Unfortunately, the anti- myc antibody generated unspecific staining of the ookinete as in both WT as well as SOAP-Apex-myc parasites we observed the same staining pattern after IFA. Therefore, we generated an additional parasite line with SOAP fused to mCherry. This allowed live cell imaging of fluorescent ookinetes to localize the protein. We could clearly see a punctuated fluorescence signal and hence we believe that SOAP-Apex-myc is most likely localized in a similar manner, since both fusion proteins do have a comparable size. This is now included in Figure 1. However, please see our answer to point 3 and to point 5 of reviewer 1 concerning the akratin-GFP localization.

3)I am not convinced by the trafficking defect. This could be because the localsation in the images are not easy to distinguish and it may be much clearer looking down the microscope. I think co-localisation with another microneme marker would go a long way and demonstrating that akratin upon mutation actually localises elsewhere is important. It is even more important since there is no phenotype in male egress, but then later in ookinetes, which is a bit surprising if this is a proper conserved trafficking motif.

Agreed, as mentioned in response to Q5 of reviewer 1, we now have evidence that the GFP fused to akratin is cleaved off in two differently generated fusion proteins, suggesting that GFP is trapped in the secretory pathway. Hence, we cannot state where akratin is localized. Evidence from *P. falciparum* akratin-mScarlet localization suggests an ER localization. This necessitated the rewriting of a large section of the respective parts and we moved the images of akratin-GFP to the supplement.

4)The candidate selection section is poorly described. A flow chart or clearer inclusion/ exclusion criteria would be useful.

Sorry for the confusion. We now tried to make this clearer. In the text it now reads as follows (line 187-190):

“To select a first candidate protein for further analyses we returned to our 81 likely micronemal candidates (Figure 1G, Table S1). Among this group we have identified 6 conserved *Plasmodium* proteins with hitherto unknown function (Figure 1G, Table S1) and selected the protein with the highest numbers of peptide hits (16), containing both a SP and at least one TMD.”

5)I understand the approach to focus on more abundant biotinylated proteins, however, I think it may not be the best approach to use peptide counting. Apex2 labelling as the authors rightfully say, is mainly based on tyrosine labelling of surface exposed areas, so the abundance of proteins in the IP will depend on accessible tyrosines, protein abundance, distance from the bait, size of the protein and how many tryptic peptides can be generated. Reproducible results between 2 conditions are more likely to show true positives and may be the best way to prioritize, or assign confidence. Also: cOuld the authors use mean intensity values for the peptides covering proteins as a metric for abundance using label free quantification? This is not a requirement but may allow quantification in a slightly better way. 
I am not sure about the Table S1 colour scheme (the legend does not explain green, purple and blue shading). Are all green ones confirmed microneme proteins? Please add a proper descripton of the table and columns.

The color scheme of Table S1 is mentioned in the last column on the table. We have now changed the colors to the colors matching Figure 1G. To better visualize our classification.

We agree that there are many ways to select a candidate protein or to make lists but ultimately the test is to target the candidates. We tried to insert a qualifying sentence but didn’t find a good place without interrupting overall textual flow. We hope this is fine with the reviewer.

6)Figure 2C and D are from PlasmoDB and should ideally not be included as figure panels. This is misleading and could either be mentioned in the text, or put into supplementary data with a clear note that the authors have not aquired these data. I would also suggest to move figures 3A-C into figure 2 and present the KO with the complementation data for a direct comparison.

Yes, that’s true. Yet for completeness and convenience we prefer to keep the information of Figure 2C/ D within the main figure. Since it already highlights expression in ookinetes and male gametes.

Instead, we have now moved Figure 1 I- K into Figure S3.

**Minor:** 

1)When the authors say "numbers of peptides identified": is this unique peptides or does it include non-unique peptides?

We have only included unique peptides.

2)Figures 1 I-K could move into supplementary as they are somewhat non-informative given the nature of BioID described in the main points.

We have now moved Figure 1 I- K into Figure S3.

3)Line 253: Whether akratin is involved in membrane lysis directly, or important for microneme secretion so this is a knock-on effect is not known. This could be added to the discussion, but there is no evidence for this statement in the results section.

Yes, we discussed initially how to handle our lack of functional understanding of akratin. We now added a sentence in the discussion to state that to find a function of akratin a pulldown of the fusion protein in *P. falciparum* could be used. We currently find it premature to speculate on where the protein functions.

4)Line 274: Refers to Figure 3F, which does not exist.

Thanks for pointing this out. We have changed it into Figure 3D.

5)Line 333: Overall I think this is a bit of an overstatement. The use of Apex2 in these conditions is definitely nice to see but for now the authors have validated none of the microneme proteins by co-localization. So we are still a bit in the dark how well the method works in terms of false positives. The targeting motif in my mind is not yet confirmed in the absence of co-localisations with other markers. An alternative explanation could be that the c-terminus of the protein is important for its function in one stage, but not another but that trafficking is not- or only marginally affected. 

Due to the presence of a large number of known micronemal proteins and the overlap with the previous study by Lal et al we are quite confident about the reliability of the method.

We are aware that proximity labelling in general also has its limitations. Due to the experimental design a common problem of the method is the detection of false positives, especially due to trafficking of the bait protein through the secretory pathway. One possibility to reduce this background is the use of Split BioID approaches as shown for ligase-based proximity labelling (Birnbaum et al). Such a tool has also been developed for Apex2 (Han et al 2019 chem biol). Yet split- Apex has not been used in *plasmodium* so far and is beyond the scope of this manuscript.

Another possibility would also be a complementary approach, using Bira* and peroxidase-based labelling fused to the same bait. Yet in ookinetes SOAP- BirA* based labelling has been unsuccessful, most likely due to long labelling times compared to Apex2.

IFA on SOAP-APEX-myc parasites using an anti-myc antibody resulted in unspecific labeling only (WT ookinetes not containing the construct showed an identical signal). Therefore, as already mentioned above, we have now generated an additional parasite line with SOAP fused to mCHerry, to monitor SOAP localization. We indeed detected a vesicular mCherry signal reminiscent to what has been published so far leading to our conclusion that SOAP-APEX-myc is most likely trafficked in a similar manner.

We have also rewritten parts of the manuscript since in the meanwhile we have evidence from western blot that the GFP fused to akratin is cleaved off. Therefore, we are unable to perform colocalization experiments. The manuscript now focuses indeed on the importance of the C- terminus.

Reviewer #2 (Significance (Required)): 

The significance of the manuscript in my mind lies in the application of Apex2 in Plasmodium parasites, which will be an advance for the field. However, we do not learn about labeling times, how short it can be so its potential is not fully looked at.

Apex2 activity has been analyzed biochemically in Lam et al Nature Methods 2014. As shown below, enzyme activity is exhausted after 1min. We hence used a slightly extended labelling time of 1.30 min as described in our methods section and didn’t test any other conditions. Labelling time is furthermore dependent on the stability of the intermediate product generated by the enzyme. Which is very short lived and known to be about 20 ms**.**

We also believe keeping labelling times rather on the short side reduces the risk of potential side effects due to oxidative stress.

The list of the putative micronemes will of course be of high interest for the community, but because of the limited validation in this study will require further validation by others. 

The identification of the dual function of this protein in transmission in egress and ookinete traversal is interesting and surely leads to further studies. The identification of a putative differential trafficking motif is intriguing, if, as stated in the major concerns, this can be validated. 

My expertise lies in Plasmodium biology with good knowledge of mass-spectrometry approaches. 

**Referees Cross-commenting** 

I agree with the assessment of the other reviewer, a slightly more detailed discussion of the hits would be desireable (exported proteins, why are they there). This could be a drawback of the system used, and mentioned. 

Western blot of the GFP is a very good idea to clarify whether the localization is maybe, in parts, GFP that is not fused to the full length protein, either by cleavage, or a breakdown product.
