# Supplementary material for: APEX-based proximity labeling in Plasmodium identifies a membrane protein with dual functions during mosquito infection
Source: PLoS Pathog. 2024 Dec 18;20(12):e1012788. doi: 10.1371/journal.ppat.1012788 (PMC11695019; doi:10.1371/journal.ppat.1012788)
Supplement: Attachment — Submitted filename: Rebuttal.docx [file ppat.1012788.s017.docx]

Prof. Dr. Kirk Deitsch

Editor - PLOS Pathogens

November 22^nd^, 2024

Rebuttal to:

„APEX-based proximity labeling in *Plasmodium* identifies a membrane protein with dual functions during mosquito infection“ - PPATHOGENS-D-24-02004

Reviewer's Responses to Questions

Part I - Summary

Reviewer #1: In this study the authors use a rapid biotynilation method to identify putative novel microneme proteins in Plasmodium berghei. After filtering of the hits and assessment of false discovery rates, they focus on a top candidate akatrin, and show that deletion of this protein causes a major defect in transmission, with a major effect in male gamete exflagellation.

Part II – Major Issues: Key Experiments Required for Acceptance

Reviewer #1: While I am happy with most of the responses of the authors, I still have a concern that would be good to clarify: in my limited understanding, there are no micronemes in gametocytes? And the authors have not provided co-localisation proof that akatrin is in the micronemes. Could you not use the Pf line to show this? This should not take too long and buy confidence that akatrin is used as a marker to validate the APEX data.

A: Yes, no micronemes in gametocytes, although there are ‘similar’ secretory vesicles such as the osmiophilic bodies. We expanded on a sentence in lines 73-75 to clarify this: “Gametocyte egress vesicles are distinct from micronemes and appear randomly distributed in the non-polarized cell, while micronemes are located at the apical end of the highly polarized migrating parasite forms”. One of the proteins that can be in both in MTRAP (our unpublished data). And yes, we would have loved to do this simple co-localization experiment, but unfortunately generated the akratin-GFP in a line (3D7) that is used as a standard by our collaborators for investigating blood stages and gametocyte development but barely makes ookinetes. Therefore, we would have to generate a new line in the strain NF54, which would cause considerable delay and resources. Also, the localization in gametocytes appears more like ER, which is why we changed the discussion in the initial revision already towards indicating this and have strengthened this more so in the current version. For example: non-modified statements lines 257-258: “This suggests that the vesicular localization of GFP results from a cleavage of the fusion protein within the ER.” and lines 266-268: “As the GFP was found in an endomembrane/vesicular location, we assume akratin is also localized in the ER and or downstream vesicles. “ and please see heavily expanded discussion in lines 395 to 434.

If it is not a microneme protein (which would be unlucky, but it could be), the phenotype data still is interesting. But the APEX data would then need to much more critically discussed. While you identify a lot of the top hits as known microneme proteins, there are also many false positives in there. You could probably try to find unifying features of the false positives (peptide count is already being one) that allows you to further increase confidence?

A: Absolutely agree on both, interesting phenotypes and possible contaminants, please see the expanded discussion from lines 395 to 434 where we expand on the limitations of our study and indicate necessary/interesting future experiments.

Part III – Minor Issues: Editorial and Data Presentation Modifications

Reviewer #1: The discussion currently lacks comments on why a microneme protein (which are normally not present in gametocytes as far as I know) shows a phenotype in exflagellation. I think this would be important to discuss.

A: Agreed, please see the expanded discussion in lines 395 to 434 as well as 464-467.
